# Interactions between a Sap Beetle, Sabal Palm, Scale Insect, Filamentous Fungi and Yeast, with Discovery of Potential Antifungal Compounds

**DOI:** 10.1371/journal.pone.0089295

**Published:** 2014-02-20

**Authors:** Andrew R. Cline, Paul E. Skelley, Scott A. Kinnee, Suzanne Rooney-Latham, Shaun L. Winterton, Christopher J. Borkent, Paolo Audisio

**Affiliations:** 1 Plant Pest Diagnostics Center, California Department of Food and Agriculture, Sacramento, California, United States of America; 2 Florida State Collection of Arthropods, Florida Department of Agriculture – DPI, Gainesville, Florida, United States of America; 3 Department of Biology and Biotechnologies ‘Charles Darwin’, Sapienza Rome University, Rome, Italy; University of Wisconsin - Madison, United States of America

## Abstract

The multi-trophic relationship between insects, yeast, and filamentous fungi is reported on sabal palm (*Sabal palmetto* (Walter) Lodd. ex Schult. & Schult. f.). Gut content analyses and observations of adult and larval feeding of the sap beetle *Brachypeplus glaber* LeConte indicate that niche partitioning of fungal food substrata occurs between adults and larvae. This is the first report of specific mycophagous niche partitioning among beetle life stages based on gut content analyses. Fungi isolated from the beetle gut of adults, larvae, and pupae include species of *Fusarium* Link, *Cladosporium* Link, and *Penicillium* Link, which were differentially ingested by larvae and adults; *Fusarium solani* and *Penicillium* species in larvae, whereas *F. oxysoproum*, F. *verticillioides*, and *Cladosporium* in adults. These data indicate the first species-level host data for *Brachypeplus* Erichson species. *Fusarium proliferatum* (Matsush.) Nirenberg was the most commonly occurring fungal gut component, being isolated from the palm as well as gut of larvae, pupae, and adults; representing a commonly shared food resource. One species of yeast, *Meyerozyma caribbica* (Vaughan-Mart. et al.) Kurtzman & Suzuki (basionym = *Pichia caribbica*), was isolated from all life stages and is likely responsible for anti-fungal properties observed in the pupae and represents a promising source of antifungal compounds; rearing and diagnostic protocols are provided to aid biomedical researchers. Feeding and cleaning behaviors are documented using time-lapse video-micrography, and discussed in a behavioral and functional morphological context. Adults spent long periods feeding, often >1/3 of the two-hour observation period. A generic adult body posture was observed during feeding, and included substrate antennation before and after ingestion. Adult grooming behaviors were manifested in distinct antennal and tarsal cleaning mechanisms. Larval behaviors were different from adults, and larvae feeding on *Fusarium* fungi immediately ceased all subsequent feeding. This is the first ethogram for any adult or larval sap beetle.

## Introduction

Resource partitioning as an advantageous life history adaptation within holometabolous insects is well-known as a mechanism for reducing competition between adults and progeny for the same food resource. However, little detailed information regarding specific empirical examples is available to support this hypothesis. In general, partitioning of resources increases survivorship of offspring and enables them to proliferate on different substrates than adults. This phenomenon has never been observed within sap beetles (for both sap beetle and host) or within any other mycophagous beetle lineage at the species level utilizing the same microhabitat.

Sap beetles (Nitidulidae) exhibit a dramatic breadth of ecological niches, more than any other beetle family, including: mycophagy, saprophagy, frugivory, anthophagy, predation, herbivory, necrophagy, pollination, and inquilinism with social Hymenoptera (bees and ants) and Isoptera (termites) [1]–[3]. Saprophagy is known from multiple lineages, including more than 20 genera from at least 6 different sap beetle subfamilies. Associations between sap beetles and soil ecology are undoubtedly important, as evidenced in the genus *Stelidota* Erichson which is found in great abundance in soil and leaf litter samples, often being the most dominant beetle component in these samples [4]. Fungal hyphae have been retrieved from the pro- and midgut of adults and larvae of this genus. The abundance and species diversity of *Stelidota*, could help provide useful insights into conservation efforts of regions and sites based on the health of the soil and litter fauna [5], [6]. Sap beetle associations with ascomycete fungi have been documented for some genera, i.e. *Glischrochilus* Reitter, *Epuraea* Erichson, *Colopterus* Erichson, and *Carpophilus* Stephens on *Ceratocystis* (Ellis and Halst.) oak wilt mats [7]–[10]. More recently, some studies have focused on fungal isolates, i.e. *Beauvaria* Vuill. from a single sap beetle species, i.e. *Meligethes aeneus* ( =  *Brassicogethes aeneus*: [11]), that were not correlated to food intake [12]. We found no sap beetle studies involving fungal associations that have focused on both the larval and adult gut.

*Brachypeplus* Erichson is a large sap beetle genus comprised of more than 100 species distributed worldwide. Highest species diversity occurs in the Old and New World tropics. New World members of *Brachypeplus* have only recently received much attention [13], [14]. Species in this genus are known to occur in association with palm vegetative or reproductive structures, specifically within the subcortical confines of dead and/or decaying plants. All species possess bodies that are highly flattened to gain entry into these confined microhabitats, and once inside persist on fungi and other components [14]. The secretive habits of the North American species, *B. glaber* LeConte, are evidenced by the fact that until D. Habeck began looking into sabal palm [(*Sabal palmetto* (Walter) Lodd. ex Schult. & Schult. f.) (Arecaceae)] inflorescences in the 1960’s and 1970’s, the species was only known from 3 specimens.

Following an initially well-defined morphology project this research evolved into a thorough evaluation of the species that led to behavioral and ecological discoveries with broader ramifications to insect biology and evolution. Herein, we provide details on the interactions of *Brachypeplus glaber* with other organisms within the confines of a sabal palm, the biogeography of the beetle and host plant, mycophagous niche partitioning between life history stages, pupal antifungal properties, laboratory rearing protocols, life table data, and natural product discovery through an understanding of a species’ natural history.

## Materials and Methods

### Specimens Examined & Biogeographical Compilation

**Figure 1:**
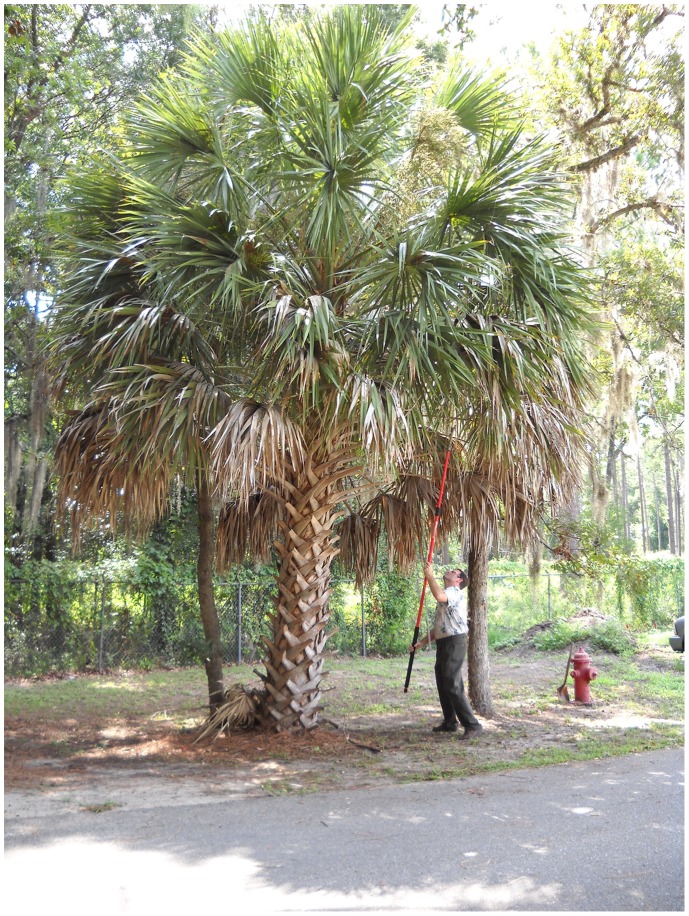
Author PES removing senescent inflorescence sheaves from a sabal palm in Gainesville, Florida.

**Figure 2:**
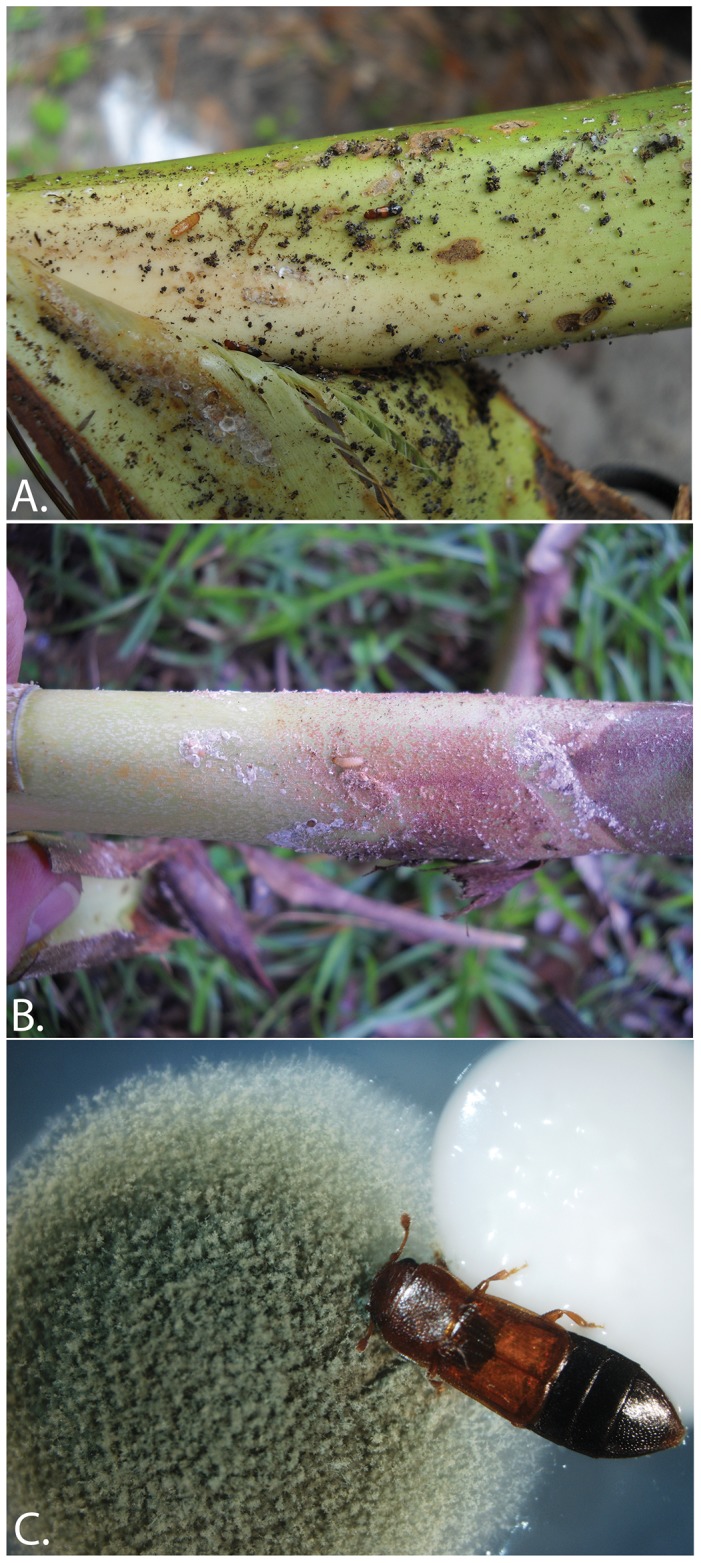
Lifestages of *B. glaber* in natural and artificial conditions. A) adults and pupae in senescent inflorescence sheave from sabal palm; B) larvae ‘in situ’ in sabal palm; C) adult feeding on *Penicillium* fungus and *Meyerozyma caribbica* yeast in Petri dish.

Adults, larvae, and pupae were obtained from live material collected by PES (Fig. 1, 2A, 2B), study of the holotype from the Museum of Comparative Zoology, and other type and non-type specimens from collections possessing New World *Brachypeplus* species. These institutes are listed below; codens prior to the institute’s name are derived from an online database of world collections (see http://hbs.bishopmuseum.org/codens/codens-inst.html) or defined here.

ARCC - Andrew R. Cline Collection, currently housed at the California State Collection of Arthropods (CSCA), Sacramento, CA, USA

BMNH - The Natural History Museum, London, England

CAS - The California Academy of Sciences, San Francisco, CA, USA

CSCA - California State Collection of Arthropods, Sacramento, CA, USA

FSCA - The Florida State Collection of Arthropods, Gainesville, FL, USA

LSAM - The Louisiana State Arthropod Museum, Baton Rouge, LA, USA

MNHN - Muséum National d’Histoire Naturelle, Paris, FRANCE

NMPC - National Museum (Natural History), Prague, CZECH REPUBLIC

TAMU - Insect Collection, Texas A&M University, College Station, TX, USA

USNM - United States National Museum, Smithsonian Institute, Washington, DC, USA

ZMHB - Museum für Naturkunde der Humboldt-Universität, Berlin, GERMANY

Locality data from collections or specimen lists were entered into an Excel file. GPS information not already provided on specimen labels was determined using online gazetteers, Google Earth, and traditional paper bound maps and atlases. All locality data was converted into degrees, minutes, and seconds for use in ArcView®. Once loaded into ArcView®, a distribution map was produced for *Brachypeplus glaber*. The *Sabal palmetto* shape file was acquired from the following website (http://esp.cr.usgs.gov/data/atlas/little/), which originated from a USDA project [15]. Digital Representations of Tree Species Range Maps were derived from Atlas of United States Trees [15], which also included data on the known distribution of *Sabal palmetto* from 1971. Other shape files were acquired from the following website (http://www.naturalearthdata.com/) and the topography was acquired from Esri®.

### Behavioral Studies

Final instar larvae and adults were kept alive for feeding and general behavioral observations. Adults were kept in Petri dishes (100×15 mm) containing either a combination of fragments of host plant material possessing live scale insects, shed scale exuviae, and active fungal growth; or artificial media and cultured fungal colonies. Each dish containing palm fragments was watered once per week to replenish moisture to the host plant and fungal substrates. Larvae were kept in standard size Petri dishes containing artificial media and cultured fungal colonies derived only from the gut contents of previously dissected adults and larvae. Using fungi from gut contents ensured that larvae would be exposed to preferred fungal substrates, and thereby increase survivability. Initial observations of adults and larvae were performed using a Nikon® SMZ 1500 stereo microscope. Some individuals were subsequently digitally recorded using a Panasonic PV-GS700 camcorder attached to a Leica MZ15 stereoscope. These digital recordings were used to determine measurements and timing of behaviors, and as a reference for functional morphology of gut action. Adult feeding behaviors were observed at 1–2 hour intervals, once a week, over a two week period. Two males and two females, four total adults, were observed with a Nikon SMZ 1500 stereo microscope to associate body postures with observed behaviors. Likewise, four total larval specimens were used to define larval behaviors. Due to the rarity of pupal forms found, only a single pupa was observed. This pupa was derived from existing larvae in the lab and was not collected from a palm in situ. All insects were denied access to food sources for one day prior to the observation period to encourage feeding.

### Dissection Protocols

Gut dissections were performed on live adults, pupae, and larvae using a Nikon® SMZ 1500 stereo microscope. Prior to dissection and between dissecting events, all dissecting tools and implements were flame sterilized to prevent contamination. For adults, the head was removed and portions of the foregut, midgut, and hindgut were subsequently removed from the body cavity with ultrafine forceps (Dumont® #5SF) and custom made angled and hook-shaped minuten pin implements attached to a nickel plated pin holder (Moria®). For larvae, the head was removed, and the thoracic and abdominal body segments splayed open via a longitudinal section down the midline of the ventrites. The fore, mid, and anterior portion of the hind gut of third instar larvae were removed using ultra-fine forceps and minuten pin tools. The dissections were performed ventral side up for all life stages to prevent unnecessary tearing of the gut wall due to its proximity to the more heavily sclerotized tergites, to assure visualization of the organs, and to preserve as much of the fragile pro, mid, and hindgut as possible. A total of 6 mature (i.e. non-teneral) adults, 6 larvae, and 1 pupae were dissected. Gut fragments were placed in distilled sterile water and processed as described below.

### Isolation and PCR Amplification of Fungal DNA

Each beetle gut was placed in a 1.5 ml tube containing 1 ml sterile distilled water. The gut was gently macerated with a sterile disposable pestle to release its contents. Aliquots (0.3 ml) of full strength solution were plated onto duplicate plates of half-strength acidified potato dextrose agar (aPDA) (BD Difco, Sparks, MD). Two serial 10-fold dilutions were also prepared and spread on duplicate plates of aPDA. Plates were incubated at 23°C under a UV light. After 7 days, morphologically distinct fungal colonies from each gut extraction were transferred onto aPDA. Genomic DNA was extracted from mycelium of 10 day-old colonies using the DNeasy Plant Kit (Qiagen, Valencia, CA). For *Fusarium* species, a portion of translation elongation factor (EF) 1a coding region and introns were amplified with primers EF-1 and EF-2 [16]. Cycling conditions included an initial denaturation for 3 min at 95°C, followed by 35 cycles of denaturation (95°C) for 20 s, annealing (58°C) for 30 s and extension (72°C) for 40 s. A final extension was performed at 72°C for 5 min. For yeasts and non-*Fusarium* filamentous fungal isolates, internal transcribed spacer region primers ITS1 and ITS4 [17] were used to amplify the ITS region including the 5.8S gene. Cycling conditions included an initial denaturation for 2 min. at 95°C, followed by 35 cycles of denaturation (94°C) for 30 s, annealing (52°C) for 30 s and extension (72°C) for 60 s. A final extension was performed at 72°C for 10 min. PCR products were purified using a QIAquick Purification Kit (Qiagen, Valencia, CA). Sequencing reactions were performed using an ABI 3730 Capillary Electrophoresis Genetic Analyzer and Big Dye Terminator V3.0 sequencing chemistry. Sequences were analyzed by performing BLAST queries in GenBank and FUSARIUM-ID [18]. All sequences were deposited in GenBank (see Table 1).

**Table 1:** Fungal isolates obtained from *Sabal palmetto* tissue and the pupa, larva and adult guts of *Brachypeplus glaber* and percent similarity to known GenBank sequences.

Fungal Species	Source	Isolate No.	AccessionNo.	% Similarity	Host/Substrate
*Cladosporium cladosporioides*	BAG	CDFA891	JX886021	100% AY463364	Unknown
*Fusarium oxysporum*	BAG	CDFA867	JX886003	100% JF740805	*Meloidogyne hapla* egg mass [Tylenchida: Heteroderidae]
*Fusarium* sp.^1^	PVT	CDFA874	JX886004	94% JF740869	
*Fusarium proliferatum* 2	BAG	CDFA865	JX886005	100% JF740773	*Zulia colombiana* adult[Hemiptera: Cercopidae]
*F. proliferatum*	PVT	CDFA873	JX886006	“”	“”
*F. proliferatum*	BP	CDFA876	JX886007	“”	“”
*F. proliferatum*	BLH	CDFA880	JX886008	“”	“”
*F. proliferatum*	BLF/BLM	CDFA882	JX886009	“”	“”
*F. proliferatum*	BLF/BLM	CDFA883	JX886010	“”	“”
*F. proliferatum*	BAG	CDFA866	JX886011	99% JF740801	*Eurygaster* sp.[Hemiptera: Scutelleridae]
*F. proliferatum*	PVT	CDFA868	JX886012	100% FJ895277	*Butia x Syagrus* palm hybrid
*F. proliferatum*	BAG	CDFA886	JX886013	99% JF740779	*Spodoptera litura* larva[Lepidoptera: Noctuidae]
*Fusarium solani*	BP	CDFA875	JX886014	100% DQ247570	Unknown
*F. solani*	BLH	CDFA877	JX886015	“”	“”
*F. solani*	BLH	CDFA878	JX886016	“”	“”
*F. solani*	BLF/BLM	CDFA879	JX886017	“”	“”
*F. solani*	BLF/BLM	CDFA881	JX886018	“”	“”
*Fusarium verticillioides* 3	BAG	CDFA862	JX886019	99% JF740717	Adult[Diptera: Bibionidae]
*F. verticillioides* 3	BAG	CDFA863	JX886020	“”	“”
*Penicillium* sp.	BLF/BLM	CDFA909	JX886022	99% DQ123635	*Coffea* spp. endophyte
*P.* sp.	BLH	CDFA916	JX886023	100% JN617707	Soil
*P.* sp.	BLF/BLM	CDFA915	–	–	–
*Meyerozyma caribbica*	BAG	CDFA887	JX886024	100% EU568999	Human nose
*M. caribbica*	BAG	CDFA905	JX886025	“”	“”
*M. caribbica*	BAG	CDFA906	–	–	–

1 Not a close match to any known *Fusarium* species.

2 Synonym = *Gibberella intermedia.*

3 Synonym = *Gibberella moniliformis.*

Source abbreviations are as follows: PVT = palm vascular tissue; BAG = beetle adult gut; BP = beetle pupa; BLH = beetle larval hindgut; BLF = beetle larval foregut; BLM = beetle larval midgut.

### Isolation and PCR Amplification of Beetle DNA

Sequence analysis was performed on fresh adult and larval specimens. Specific methods were previously published in a companion morphology paper [14].

## Results

### Behavioral Observations

#### Adult

Upon introduction onto a Petri dish containing fungal components, one male beetle fed for approximately 20 min., took a short 2–3 min. break, and resumed feeding for another 22 min, finally ending in a period of rest with the body still. A second male initially fed for approximately 8 min, took a 12 min. break, and resumed feeding for another 24 min, ending in a period of rest. One female initially fed for approximately 8 min. then walked around before becoming stationary for the rest of the 2 hr. observation period. A second female likewise fed for a brief period of 7 min then walked to the edge of the Petri dish and remained motionless for the remaining 1 hr observation period.

Both the first male and second female were observed feeding on the shed exuviae of the scale insect, *Comstockiella sabalis* Comstock (Diaspididae), during the observation period. In these instances, the shed exuvium was completely ingested, as well as *Fusarium* (sp. 1) hyphae present on the exuviae. The ingestion was completed within one min. for both beetles, and there was no correlated body posture or other functional morphological manifestation during the process. Feeding events that included exuviae consumption ceased all other feeding activities on fungi. Both beetles consumed the shed exuviae in the same manner as fungal substrates, i.e. no observed functional morphological or behavioral differences.

Both male beetles were observed at separate time periods feeding in damp areas of the Petri dish that were partially submerged in drops of water. Feeding within submerged areas continued in the same manner within water as it did when the beetle was not submerged. One male consistently extruded its genitalic capsule (i.e. ventrite 8 and tergite 8) slightly outside of the body cavity during feeding. The median lobe and tegmen, however remained within the capsule and were never extruded. Another male, during a short break in feeding, flexed its abdomen in an upward bow shape and partially unfurled its mesothoracic flight wings. This occurred several times during short feeding breaks. Several minutes (∼14 min) after cessation of feeding activities, this male was the only beetle observed to produce fecal material. The second male specimen was also observed feeding on the cast larval head capsule of a conspecific; however the head capsule was not fully consumed. All four test specimens were observed feeding on fungal particles that had accumulated on their conspecific’s dorsum while in the Petri dish.

Adult functional morphology during feeding was expressed with a somewhat generic body posture in which the legs remained stationary to the sides, while the beetle fed from directly in front of its head, i.e. prognathous style. The antennae only infrequently tapped the substrate (every 10–15 s), and were typically held in a position 90° to 120° forward from a longitudinal transect down the midline of the body, and roughly 10° to 30° degrees superior to a transverse sagittal plane of the body. The mandibles and maxillae moved in concert with each other, and the terminal maxillary palpomere extended beyond the mandibular apices during periods of initial food consumption, and was the first mouthpart sclerite to come into direct contact with the food source. This probing by the terminal palpomere likely coincides with determination of food source suitability, as the palpomere apex is completely covered with sensory setae. Food particles were often manipulated and reoriented, via a combination of movements of the mandibles and maxillae, to more easily fit within the oral cavity. Due to the relatively light sclerotization of the beetle head and pronotum (which made the beetle somewhat transparent) as well as the use of both ring lights and directional fiber optic light, visualization of the foregut was possible at certain angles. Food items entering the oral cavity typically spent 1–2 s in the esophagus and oral cavity.

Cleaning behaviors expressed by all adults consisted of tarsal and antennal club grooming. Cleaning of the antennal club was accomplished by two different mechanisms. One mechanism involved the terminal two tarsomeres (including the claw) of the protarsi grasping one antenna from above and sliding the antenna through the tarsomeres in an anterior/inferior motion. The other mechanism involved using one or the other protarsi to push the antenna (from segment 6–7) between the mandibles and maxillae in an anterior/inferior (AI) motion or an anterior/superior (AS) motion such that the entire antennal club is brought between the mouthparts from either above or below the head. The frequency of occurrence of the AS motion is far less than the AI motion, such that the antennal club was 4–5 times more likely to be cleaned in a downward motion than upward motion of the antenna through the mouthparts (n = 14, AI = 12, AS = 2). All antennal cleaning behaviors were expressed in both genders, with no particular behavior relegated to one sex or the other.

Tarsomere cleaning was observed for each leg. Protarsomeres were cleaned via three different mechanisms. One method involved bringing the terminal 3 tarsomeres, including the claw, between the mandibles and maxillae in a superior to inferior motion (i.e. mouthparts clean tarsi). Another method included rubbing the protarsomeres of the right and left legs together in a vigorous back and forth motion (i.e. ‘hand washing’ motion to clean tarsi of same body segment) beneath the head. The third method was to clean the protarsomeres with the mesotarsomeres of the corresponding side of the body in a vigorous back and forth motion similar to that of the protarsi to protarsi motion above (i.e. ‘hand washing’ motion with tarsi of different body segments). Protarsal to mesotarsal cleaning occurred on both the left and right sides of the body. The mesotarsi were cleaned on the metatibial comb on the corresponding side of the body in a superior to inferior motion from proximal to distal regions of the comb on the metatibiae. Mesotarsi and metarsi were also cleaned by a similar hand washing motion of tarsi from the same body segment beneath the body proper. All tarsal cleaning behaviors were expressed in both genders, with no particular behavior relegated to one sex or the other.

General behaviors expressed by adults were observed in Petri dishes containing cultured fungi (Fig. 2C), as well as Petri dishes containing palmetto fragments with fungal and other debris present. Most of the observation periods were spent either feeding or remaining stationary (>95%), with little time spent walking around the Petri dish. During stationary periods, the beetle’s antennae often vibrated in short frequency such that under high-power magnification the antennae appeared to shake. This may be a mechanism for breaking up laminar air flow around the antennae, which creates turbulence and thereby increases the likelihood of detecting volatiles in the surrounding air through sensory receptors on the antennal club segments (i.e. segments 9, 10, and 11). No air-flow tests were performed to confirm this hypothesis, rather this assertion is based solely on behavioral observations and the known morphological adaptations of the Nitidulidae antenna.

#### Larvae

Feeding behaviors expressed by larvae were typically short-lived, lasting only 3–4 min. Of the four larvae observed, one larva fed on *Fusarium* sp. 1 for 8 min. As with adults, food particles remained in the foregut for a very short period of time, typically never more than 2–3 s. Larvae preferentially fed on *Fusarium* Link and *Penicillium* Link species, with the largest proportion of time spent feeding on *Fusarium* hyphae (approximately 2∶1 preference duration, 476 s on *Fusarium* to 230 s on *Penicillium*, n = 4). Feeding progressed in a prognathous manner as in adults, however the mandibles and maxillae in the larvae extended forward to a similar length to come in contact with the substrate at the same time/place. Any consumption of *Fusarium* hyphae by larval specimens caused subsequent cessation of all feeding activities. No cleaning behaviors were observed in any of the larval specimens. Larvae spent less time in ambulatory movement than adults, and most time was spent either motionless or feeding (>99% of observation time). General behaviors expressed by larvae were observed solely in Petri dishes containing fungi that were cultured from larval gut contents. No larval behaviors were recorded on palms or palm cuttings.

#### Pupae

Pupal behavior was observed in a Petri dish containing cultured fungi, i.e. *Fusarium proliferatum*, *Fusarium solani* (Mart.) Sacc., and *Penicillium*. The beetle pupated venter side down with the last larval skin pushed to the apex of the pupal abdomen, but still attached to terminal abdominal tergite and ventrite. Activity was expressed in the flexion of the abdomen in a curling manner towards the ventral surface of the head and thorax. In a 36 hr period, the newly developed pupa discarded the last larval skin and moved approximately 1 cm from it. In the subsequent 10 hours the pupa flipped from a ventral side down posture with the abdomen flexed and the body in an inverted “U” position to a prostrate flattened posture with ventral side up. Although perhaps insignificant in distance, the movement away from the final cast larval skin may be biologically important. Specifically, the observed occurrence of adults feeding on cast larval skin may warrant quick disposal and dispersal away from the shed skin to avoid potential predation (whether inadvertent or not) by conspecific adults. Also, within the roughly two day period from pupal emergence to a flattened prostrate position, the final cast larval skin began producing copious *Fusarium* hyphae and spores. Thus, movement away from the final cast larval skin may have provided the newly emerged pupa an opportunity to distance itself from fungi that had once been consumed and/or impregnated the outer cuticle, and which could potentially consume the quiescent pupa. This behavior was not observed within the confines of the sabal palm inflorescence where flexion and curling may be severely restricted depending on the degree of separation of the inflorescence sheaves. Pupal mobility is not unknown in Coleoptera [19], but has never been previously observed in Nitidulidae.

One chemically mediated pupal behavior was observed via the secretion of an antifungal agent to the immediate surrounding Petri dish environment. Approximately 10 days after the transition from larva to pupa the Petri dish was reexamined and the pupa remained viable, however the Petri dish had begun to be over taken by fungal growth, except for a distinct fungal free zone immediately around the pupa (Fig. 4). Although some fungal hyphae permeated the media underneath the pupa, no surface hyphae or fruiting bodies breached came in contact with the pupa.

### Fungal and Yeast Components of the Gut

The internal gut flora that was determined for both adults and larvae is categorized below. Each fungus or yeast species is treated separately. Table 1 should be used as a reference for each component. Sequences of the translation elongation factor (EF) gene or ITS regions for most isolates were deposited into GenBank. Most of the filamentous fungi isolated from the gut flora were members of the genus *Fusarium*. *Fusarium* species include plant and animal pathogens, common soil and environmental inhabitants, and important mycotoxin producers. The use of alpha elongation factor gene sequencing in *Fusarium* identification is typically necessary as morphological characters alone are often not sufficient for accurate identification.

*Fusarium proliferatum* (Matsush.) Nirenberg, also known as *Gibberella intermedia* (Kuhlmann) Samuels, was the most commonly isolated fungus in our study. This species was isolated from every sample, including adult beetle gut, pupal gut, larval gut, and hyphae directly plated from live palm samples. *Fusarium proliferatum* exhibits a worldwide distribution and causes many plant diseases, particularly on monocots, including rice, maize, onions, garlic and palm [20]. This species is also an important producer of the mycotoxins fumonisin B1, fusaproliferin, beauvericin and fusaric acid [20]. Most sequences of *F. proliferatum* isolates (CDFA865, 873, 876, 880, 882, and 883) obtained from beetle guts were identical and most closely matched *F. proliferatum* sequences obtained from other insects (NRRL 52687, NRRL25103) [21].

*Fusarium verticillioides* (Sacc.) Nirenberg, also known as *Gibberella moniliformis* Wineland, was isolated from adult beetle guts and live palm samples. This fungus is particularly important on corn as it causes a pervasive stalk and cob rot resulting in significant yield reductions and grain quality [22]. *Fusarium verticillioides* is a widely recognized mycotoxin producer, especially fumonisins [22], [23], as well as being responsible for ∼20% of *Fusarium* infection in humans [24]. Many insect taxa play a major role in dispersal of this pathogen and subsequent disease and mycotoxin levels [25]. Volatile compounds that are produced by *F. verticilloides* are thought to be attractants to many insect species (Bartlet et al 1999) [26]. The two sequences of *F. verticillioides* obtained in this study (CDFA 862 and 863) most closely matched sequences isolated from an adult flies in the family Bibionidae (NRRL 25087) [21].

*Fusarium solani* was isolated from all larvae and pupae, but was not isolated from guts of any adult beetles (CDFA875, 877, 878, 879 and 881). This species exhibits a worldwide distribution and, in addition to being a common soil inhabitant, is pathogenic on many host plants including beans, citrus, squash and peppers [22]. This species also accounts for most (∼50%) of the *Fusarium* infections in humans [24], [27].

In addition to *Fusarium proliferatum*, another *Fusarium* species was directly isolated from mycelia on palm tissue (CDFA874). Blast sequence comparison demonstrated a 94% similarity to isolates of *F. concolor* Reinking (NRRL 01847 and NRRL 13459) previously deposited in FUSARIUM-ID database. The relatively low similarity of this isolate to other known species suggests it may prove to be a novel species. However, since it was only cultured from palm tissue, it doesn’t appear to be an important food source for *Brachypeplus glaber* individuals in this study.

Two other filamentous, non-*Fusarium* fungi were also cultured in this study. *Cladosporium cladosporioides* (Fresen.) G.A. de Vries was isolated from adult beetle guts that fed from the live cut palm pieces (CDFA891), while *Penicillium* species were isolated from guts of all larvae (CDFA909, 916 and 915). Species of *Cladosporium* Link and *Penicillium* are some of the most common and cosmopolitan fungi worldwide.

The yeast *Meyerozyma caribbica* (Vaughan-Mart., Kurtzman, S.A. Mey. & E.B. O’Neill) Kurtzman & M. Suzuki (anmorph = *Candida fermentati*), was isolated from pupae, larvae and adult beetles from live palm samples. *Meyerozyma caribbica* is commonly encountered in both natural and clinical environments [28; Vaughn-Martini et al. 2005 29, 30]. *Meyerozyma caribbica*, and the closely related species *M. guillermondii* (Wick.) Kurtzman & M. Suzuki, are believed to form endosymbiotic relationships with many insect species. *Meyerozyma caribbica* has been cultured from the guts of nitidulid and scarabaeid beetles [31], as well as the fungal combs of fungus growing termites [32]. This species may play an ecological role in xylose fermentation of wood-ingesting beetles as many isolates have been shown to be high xylitol producers [31], [33]. Both are considered antagonistic yeasts, and have been used as biological control agents for postharvest fungal fruit decays [34]–[37].

### Biogeography, Natural History, and Biology of *Brachypeplus glaber*

**Figure 3:**
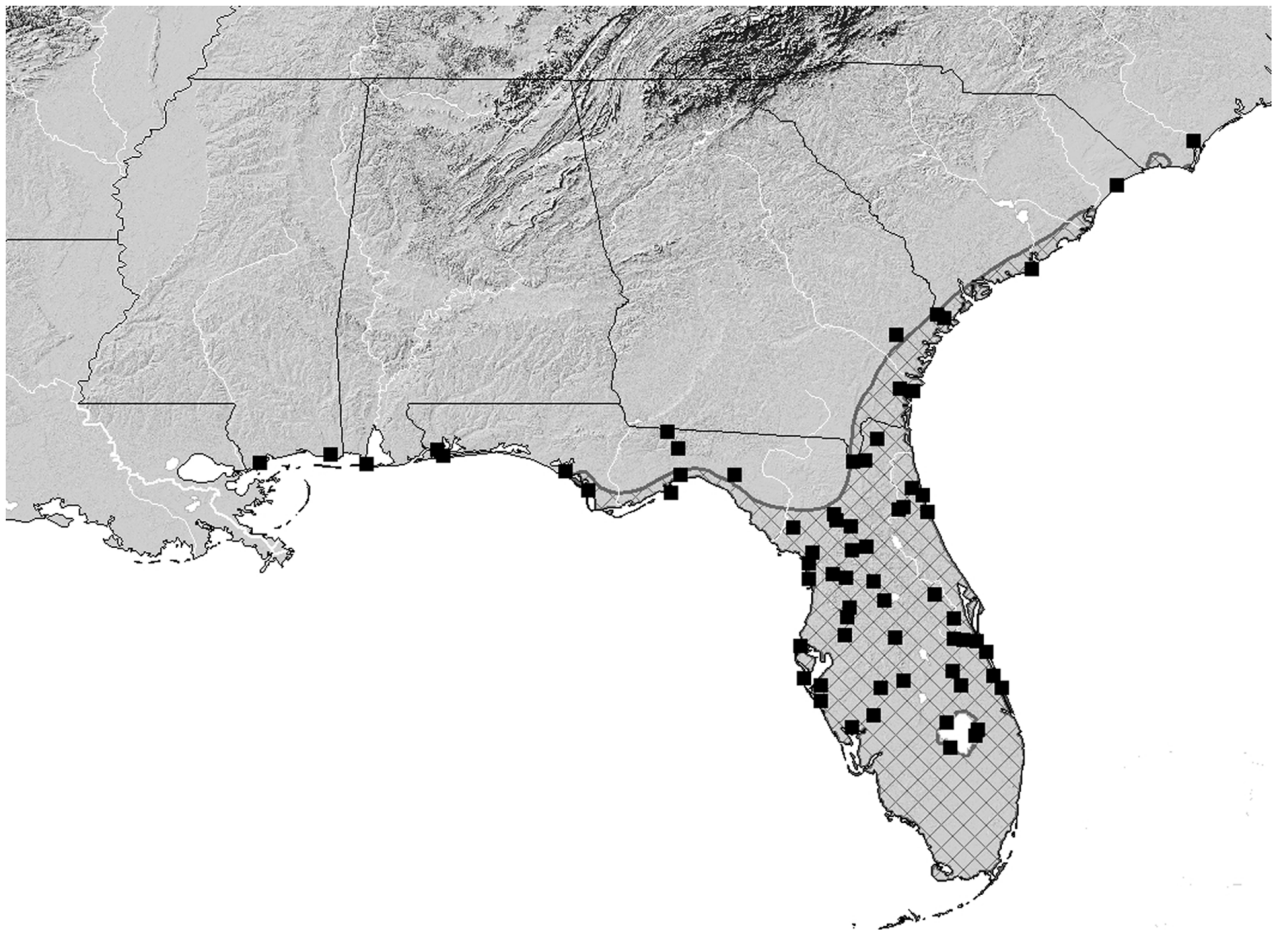
Known distribution of *B. glaber* as denoted by black squares. Meshed area indicates the native distribution of *Sabal palmetto*.

The distribution of *B. glaber* coincides with the native, and potentially part of the introduced range, of *Sabal palmetto*, i.e. the sabal palm (Fig. 3). To date, very few adult specimens have been collected that were not associated in some way with inflorescences of *S. palmetto*. Hamilton [38] collected five specimens from a “dead, standing pine, with *Cossonus impressifrons*” at Lake Worth, Florida. *Cossonus impressifrons* Boheman (Curculionidae) is a subcortical weevil that is often found under the bark of various conifers and hardwoods, and likely has no significance to the occurrence of *B. glaber*. Other than these specimens taken under pine bark, most other adult specimens with associated biological data are from the flower stalks of *S. palmetto*. The presence of *B. glaber* in the subcortical habitat of the pine tree was either an accidental occurrence, misidentification, or was indicative of a refuge for the beetle that provided shelter a food source, or both. The authors were unable to locate Hamilton’s specimens to corroborate the identification, and thus misidentification may also be possible.

**Figure 4:**
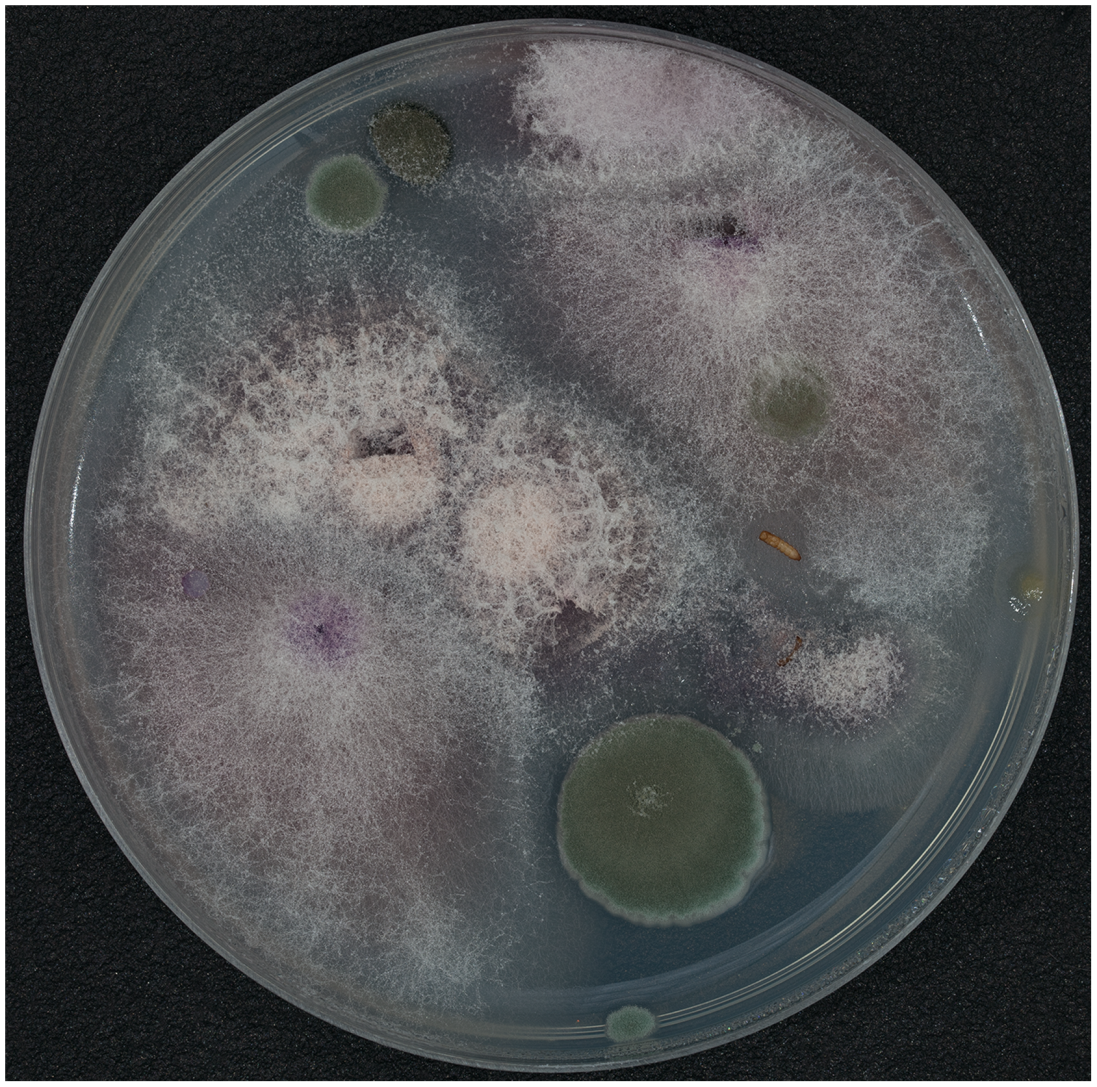
Overgrown Petri dish showing ring (zone of inhibition) around pupa with no fungal growth.

The other rare occurrences of *B. glaber* away from *S. palmetto*, e.g. in flight intercept traps, are likely the result of dispersal activities of the adults to other suitable *S. palmetto* hosts that are in flower, and may be accounted for as incidental collections. Furthermore, no immature stages of this beetle have been collected outside the confines of sheaves of mature inflorescence stalks of this palm species. Therefore, we suggest that although these beetles are not known to ingest host plant material, they may require the specific microhabitat established by the inflorescence stalks of *S. palmetto* to complete their life cycle (i.e. development of immature stages). Recent collections of *B. glaber* in Florida further support that the host plant may not be the sole prerequisite for successful development. These recent *B. glaber* collections have been in association with the presence of a diaspidid scale, *Comstockiella sabalis* (Comstock). *Comstockiella sabalis*, or palmetto scale, is present in the southern United States along the Gulf Coast to Mexico, and is also present in parts of the Caribbean, effectively encompassing the same distribution as *S. palmetto* and *S. mexicana* Mart.; however it is not necessarily host specific to these two species [39]. The life cycle of this scale is complex, and often is associated with the presence of a fungus [39]; unfortunately the identity of this fungus remains unknown. One of the many peculiarities of this scale insect is that it does not incorporate the shed skins into its scale cover and often they are discarded. This latter phenomenon may be important for *B. glaber*, which was observed feeding on the discarded shed exuvial remnants. These shed skins likely provide the beetle not only with a source of proteinaceous matter, but may also be the site for fungal and/or yeast development within the inflorescence microhabitat. These shed skins are essentially a meshwork of chitin, wax, and other organic matter that would effectively retain moisture and thus provide a suitable substrate for fungal/yeast development within the confines of the inflorescence stalk. Thus, consuming the shed skins may provide the beetle not only with a source of additional dietary protein, but may also be a key component for ingestion of their gut flora.

In the 1960s, *B. glaber* was reared under laboratory conditions by Dr. Dale Habeck at the University of Florida. Habeck placed numerous adults on small pieces of sabal palm flower stalks in 1 oz. plastic cups. Each cup contained about 0.25 inch of Shorey and Hale media [40] to provide moisture. The adults were subsequently moved to new containers daily to obtain life history data. From Dale’s extensive notes we provide the following life table information for *B. glaber*. Average length of entire life cycle (n = 45) from egg to adult was 35 days (range = 26–45 days). In two observed hatchings, the duration of the egg stage was four days, which is similar to that of other known Nitidulidae [3]. The average larval development from egg to pupa (n = 8) took an average of 27.9 days (range 21–36 days). Like most other Nitidulidae, members of *Brachypeplus* likely pass through three larval instars [3]. The average pupal developmental phase (n = 27) lasted an average of 7.9 days (range 4–13 days). This rather rapid pupation process is typical of most multivoltine sap beetle species [3], and *B. glaber* does exhibit multiple generations per year. Rapid pupation in a quickly decaying substrate that is already filled with varied fungal components may be advantageous for successful survivorship by avoiding becoming food for fungi. This particular strategy would enable the individual to remain protected in an enclosed, albeit decomposing, refugium until the mobile adult phase.

Eggs are laid singly or in small groups (up to five observed) in the green areas of the inflorescence stalks that are just beginning to mold. Small, presumably first instar, larvae are often found in the same area as the eggs. Larger, presumably second and third instar, larvae and pupae are found only in the moldier, decaying areas of the inflorescence stalk. When inflorescence stalks first appear, only adults are found and no immature stages. As the stalks develop and become larger and older, with the tips of the sheaths beginning to turn brown, eggs and small larvae begin to appear. As the stalks gradually senesce, becoming progressively more infested with molds, mature larvae and pupae can be found. Adults are found year round in the Gainesville area, and only adults are known to overwinter in this area. Collection label data support this phenomenon in general throughout the species range (see Appendix S1).

Gut content analyses (see Table 1) suggest that adults are primarily feeding on species of *Fusarium* (specifically *F. oxysporum* E.F. Sm. & Swingle and *F. verticilliodes* (Sacc.) Nirenberg) and *Cladosporium cladosporioides*. In contrast, larval gut contents and behavioral studies revealed a preference for different *Fusarium* species [i.e. group 1 of *F. proliferatum* (Matsush.) Nirenberg ex Gerlach & Nirenberg, *F. solani*, and *F*. sp. 1] as well as *Penicillium* species. Very few fungal components were found to be similar between the guts of adults and larvae, however, all life stages (except the egg, which was not analyzed) were found to contain the yeast *M. caribbica*.

## Discussion

The observed associations of the beetle, scale insect, plant, fungi, and yeast of this system yield more questions than answers. Clear roles for each of the constituents of the system remain somewhat obscured. However, the following is what we have observed, know, or presently suspect. With few exceptions, the beetles have been found only in sheaths of sabal palm flower stalks which are also occupied by a scale insect. Based on Dale Habeck’s previous notes, in conjunction with our own collecting and observations presented here, *B. glaber* apparently occurs only within the confined spaces of sabal palm flower stalks. Additional palm species have been sampled, but not extensively. However, considering the amount of effort spent by other researchers collecting on palm and palm flowers, it is obvious that these beetles can be easily overlooked and are found only if the collectors are looking in the appropriate niche.

We attempted to rear additional *B. glaber* specimens for deposition in museums. Palm stalks were cut into small sections and placed in a 0.5 m^2^ cardboard box. This box was then sealed with tape and a glass emergence jar placed in a hole on the side. Emergence chambers such as this are used to collect insects as they emerge from hosts and try to escape by moving to the light visible through the glass jar. After several months with no *B. glaber* present in the emergence jar, the rearing effort was considered a failure and the box was broken down for disposal. At that time, several hundred dead adults were found in the layers of the box and stuck to the tape. This phenomenon emphasizes that beetles like *B. glaber* can be present in large numbers within a niche, and rarely if ever collected in nearby situations even on the same structure. Without thorough sampling of a suspected niche, secretive beetles such as this may be easily overlooked, even by experienced collectors. This result also suggests that these beetles exhibit negative phototaxy, which would be advantageous for exploiting the confines of inflorescence sheaves.

In support of the observation of a secretive existence, *B. glaber* has never been collected (to the best of our knowledge) by anyone working with insects that pollinate sabal palm flowers. We have thoroughly examined sabal, and other palm species flowers looking for weevils, cerambycid beetles, and other interesting insects, but no *B. glaber* have been collected. Simply collecting on flowers is not enough to find *B. glaber*. The palm stalks must be peeled, layer by layer, looking closely between the sheaths. Niche specialization is only one reason *B. glaber* is rarely encountered. Sabal palms bloom only after they have matured, and in many cases these blooms are well out of reach of any collector even when using long-handled nets. Thus, the majority of *B. glaber* populations spend their lives well out of reach of researchers.

The armored scale insect *C. sabalis* (Diaspididae) is specific to sabal palms, frequently abundant on the host, and commonly found with *B. glaber* in the flower stalks. Being an armored scale, they secrete little or no honey-dew and produce very little wax. There is no evidence that *B. glaber* feeds on living scales, however, they do feed on shed skins. The chemical and nutritional profile of the shed skins remains unknown, however further examination of their make-up may provide insight into interesting components of the beetle diet and a mechanism for obtaining otherwise rarely encountered food sources (i.e. proteins, vitamins, and co-vitamins).

The fungi and yeasts isolated from the beetles that grow on the palm surface are known to occur in other places and there are likely other fungi and yeasts that could occur in sabal palms. Our sampling of fungi and yeasts was from a single locality, and it is possible that different fungi or yeasts are used by *B. glaber* in other localities. Additional sampling is required to confirm our results over the beetle’s known range, but is beyond the scope of this paper. However, as noted by Suh and Blackwell [41], often there is only one yeast species isolated from a single beetle species regardless of range. Thus, the likelihood of the yeast component changing across the range of *B. glaber* is minimal. At present, it appears that *B. glaber* is a niche specialist on sabal palms, where the niche is arboreal flattened cavities in living flower stalks that contain certain filamentous fungi and the yeast *M. caribbica*. Additional sampling and study of other palms in Florida will help solidify the hypotheses that *B. glaber* is restricted to the sabal palm, and may help explain why. Lachance et al. [42] demonstrated that anthophilous sap beetles often transfer specific yeasts as potential food substrates and that some biogeographical implications can be traced as a result. Their study included *Conotelus* Erichson, a genus related to *Brachypeplus* within the subfamily Cillaeinae. Our results indicate that yeasts associated with *Brachypeplus* are different from those of *Conotelus*, and no *Conotelus* in their study harbored any *Meyerozyma* species. Further research on yeast associations in sap beetle guts and a robust phylogeny of sap beetles, may prove useful in understanding shifts from anthophily to fungivory or vice versa (see also scenarios discussed by Kirejtshuk [43]).

Collection of sap beetles on other fermenting sources indicated that low numbers of specimens visit areas with *Penicillium* present [7]. However, this study only focused on adult sap beetles, and the presence of *Penicillium* in all larval guts in our study may indicate that these fungi are preferentially used by larvae for development and are not needed by adults. Adult and larval physiology are dramatically different in holometabolous insects, and *Penicillium* may provide developing larvae with the requisite materials needed for successful development. Likewise certain species of *Fusarium* may possess proportionately more essential amino acids, vitamins, sterols, and other nutrients that other species of *Fusarium* do not possess for optimal larval development, or conversely for adult gamete development during oogenesis and spermatogenesis. Thus, larvae and adults may be preferentially selecting different fungal species based on different dietary and developmental needs. These and other physiological requirements may ultimately be the driving force of resource partitioning between adults and larvae in *B. glaber*. The role of the ingested scale insect shed exuviae is unknown but may be providing additional nutrients to adults as well.

### Life History Stage Associations

Immature forms of Nitidulidae are mostly known from agriculturally important genera such as *Brassicogethes* Audisio and Cline, *Carpophilus* Stephens, *Stelidota* Erichson, and *Glischrochilus* Reitter. The remainder of the taxa, which often exhibit cryptic lifestyles, remain little known. Larvae of some species, such as members of *Psilopyga* LeConte, occur on ephemeral substrates such as stinkhorn fungi; and many other genera, e.g. *Cyllodes* Erichson, *Pallodes* Erichson, and *Apsectochlius* Reitter, occur on relatively ephemeral Agaricales mushrooms. Others, i.e. *Thalycra* Erichson, feed on hypogean Hymenogastrales fungi such as *Rhizopogon* Fr. Others such as *Xenostrongylus* Wollaston are leaf miners as larvae. However, many larvae, like *Brachypeplus*, occur in subcortical conditions completely obscured from view where they likely persist on wood decaying fungi and yeasts. As noted in Cline et al. [14], the rearing and molecular methods used for associating life stages of *B. glaber* not only provide a 100% reliable means for discerning this species, but establishes a protocol for other sap beetle workers, and beetle workers in general, to begin determining and describing immature stages.

### Biomedical Implications

The biomedical aspects of this research show potential for bio-prospecting within the southeastern United States. There appear to be antifungal properties of the pupal stages of *B. glaber*, and these properties may extend to larval and adult stages as well (though not tested here). Our results indicated that *Fusarium*, *Cladosporium* and *Penicillium* were all negatively affected an antifungal compound associated with the pupal stage, as evidenced by a distinct zone of inhibition extending several millimeters outward from the pupal body in all directions. No fruiting bodies or hyphae from any of the fungi penetrated the zone of inhibition. The affected fungi are representatives of three disparately related fungal lineages, and all are of medical importance as they are known to cause a variety of infections, particularly in immune-compromised patients [24]. For example, two of the *Fusarium* species we found (*F. solani* and *F. verticillioides*) are responsible for 70% of *Fusarium* infections [24] and are notoriously resistant to current antifungal drugs [27]. Secondly, the *Cladosporium* species we found (*C. cladosporioides*) was recently found to cause skin lesions in humans [44]. Though most *Penicillium* species do not infect humans, *P. marneffei* Segretain causes penicilliosis, particularly in immune-compromised individuals [24]. Both *Cladosporium* and *Penicillium* species are commonly found in homes as ‘black-mold’ and their spores are thought to be a significant contributor to the development of asthma in children [45], [46]. Development of the broad spectrum antifungal compound(s) present in *B. glaber* could provide a new method of treatment, and may open up avenues of research into a potential new family of antifungal chemicals.

At this time it is unclear whether the antifungal compound(s) are secreted by the pupa or an associated microorganism. The yeasts associated with the beetles may play a role in the fungal suppression we observed, as may bacteria (though none were observed). The yeast *Meyerozyma caribbica* seems the most likely candidate as the related *Meyerozyma guilliermondii* has been shown to significantly reduce the amount of infection by the post-harvest green and blue mold citrus pathogens, *Penicillium digitatum* (Pers.) Sacc. and *P. italicum* Stoll respectively [34], [35]. Likewise, it has also shown to have antifungal properties against gray mold of strawberries caused by *Botrytis cinerea* Pers. [36], [37]. In both instances, cells of *M. guillermondii* were shown to directly attach to hyphae of the plant pathogen and, in the case of gray mold, to secrete cell wall degrading enzymes against the pathogen [35], [36]. Various bacteria are also known to provide antifungal and antimicrobial defenses when living symbiotically with insects [47, 48; 49; 50; 51; Kaltenpoth 2009). Though no colonies of bacteria were observed in our study, this possibility should be explored further.

Sabal palms are easily grown in cultivated situations as ornamental plants in urban landscapes, therefore nursery microhabitats for mass-rearing *B. glaber* are well known. Likewise, rearing *B. glaber* on a combination of artificial diet and palm cuttings has already been demonstrated by Habeck in the 1960s, and was detailed herein. Therefore, rearing *B. glaber* in laboratory situations is possible; however acquisition of yeast or other anti-fungal agent may not be possible if beetles are solely reared on artificial diets. The methods above also clarify the culturing and DNA sequencing of the fungal and yeast components, which provide reliable identification of these fungi. We are currently applying these methods to develop a protocol for extracting and isolating the antifungal secretion from the immature stages of the beetle. Once the compound is isolated, simple trials against pure cultures of known fungal pathogens will ensue to determine the compound’s efficacy. These results will be discussed in an upcoming paper.

## Conclusions

An understanding of the natural history of insects and their kin may indeed provide great advances in our understanding of the micro and nanobiological realms. Although the small size scale of insects, fungi, and yeast have previously deterred investigations into their biology, the ever-increasing sensitivity of molecular methods now allow for greater and more complex studies of these organisms. Trips to remote rainforests may not be necessary to identify, characterize, and mass produce biomedical compounds for human use. However, an intimate knowledge of the biology of the target organism is the requisite first step to achieving the discovery of natural products from any ecosystem. We hope this research leads to other fruitful discoveries from insect-plant-fungal interactions and/or the interesting life history biology of insects in our own backyard.

## Supporting Information

Appendix S1 (DOCX)
