# Supplementary material for: Interactions between a Sap Beetle, Sabal Palm, Scale Insect, Filamentous Fungi and Yeast, with Discovery of Potential Antifungal Compounds
Source: PLoS One. 2014 Feb 20;9(2):e89295. doi: 10.1371/journal.pone.0089295 (PMC3930711; doi:10.1371/journal.pone.0089295)
Supplement: Appendix S1 — (DOCX) [file pone.0089295.s001.docx]

**APPENDIX 1.** Non-type material used for analysis and production of distribution map.

The following conventions are used for labels when possible: / = label break, L = larval records, P = pupal records. Acronyms in brackets are museums/institutions where specimens are currently housed.

Other Material Examined.

ALABAMA:

- Mobile Co. / Dauphin Island / 20-XI-1986, D.H. Habeck / *Sabal palmetto* flower stalk [ARCC]

FLORIDA:

- Alachua County: Gainesville / 29°38.1’N 82°22.3’W / ex. *Sabal palmetto* flower stalks / 11 Nov 1975, coll. D. Habeck [LSAM]

- Alachua County: Gainesville, Doyle Conner Building, 23-VIII-1991, P.E. Skelley, cabbage palm flower stalk [FSCA]

- Alachua County: Gainesville, Doyle Conner Building, 23-VIII-1989, P.E. Skelley, cabbage palm flower stalk [FSCA]

- Alachua County: Gainesville, 23-VIII-1991, M.C. Thomas, inflorescence of *Sabal palmetto* [FSCA]

- Alachua County: Gainesville, FDACS-DPI, 1911 SW 34^th^ St., 5-9-VIII-2011, M. & P. Skelley, M.C. Thomas, sabal palmetto flower spike [FSCA, ARCC] L,P

- Alachua County: Gainesville, 23-VIII-1991, M.C. Thomas, inflorescence of *Sabal palmetto* [FSCA]

- Alachua Co., Gainesville, 4-X-71, J. B. McGowan [FSCA]

- Alachua County: Gainesville, 11-XII-1975, D.H. Habeck, cabbage palm flower stalks, A-1659 [FSCA] L,P

- Alachua County: Gainesville, 11-XI-1975, D.H. Habeck, cabbage palm flower stalks [FSCA, ARCC] L

- Alachua County: Gainesville, V-VI-1968, D. Wojcik & D.H. Habeck, cabbage palm flower stems [FSCA, ARCC] L,P

- Brevard Co., 5m E of Brevard/Osceola 1, Hwy 192, 29-VII-1985, Habeck & Thompson, cabbage palm flower stalks [FSCA]

- Brevard Co., ca. 15 m. E. Brevard/Osceola Co. Ln. Hwy 192, Habeck & Thompson, cabbage palm flower stalks [FSCA]

- Escambia Co., Pensacola, 19-VII-1968, D.H.Habeck, R. E. Waites, *Sabal palmetto* flower stems [FSCA]

- Gulf County: Beacon Hill / 6 July 1968 / D.H. Habeck collector / *Sabal palmetto* flower stems - [CAS]

- Gulf Co., Beacon Hill, 6-VII-68, D.H. Habeck, *Sabal palmetto* flower stems [FSCA]

- Leon Co., Tallahassee, motel, 11-IX-1974 [FSCA] L,P

- Osceola Co., 2 m W of Brevard/Osceola 1, Hwy 192, 29-VII-1985, Habeck & Thompson, cabbage palm flower stalks [FSCA]

- Osceola Co., Yeehaw Jct., 15-III-1970, D.H. Habeck, *Sabal palmetto* flower stems [FSCA]

- Pinellas Co., Ft. DeSoto St. Park, 22-X-1971, D.H. Habeck, *Sabal palmetto* flower stems [FSCA]

- Pinellas County, Dunedin [MCZ]

- St. Johns Co., DeeDot Ranch, 12-X-1972, DP Wojcik, *Sabal palmetto* flower stalks [FSCA]

- St. Johns, Anastasia State Pk., 26-VIII-1972, D. P. Wojcik, *Sabal palmetto* flower stems [FSCA]

- St. Johns, Anastasia State Park, 26-VI-1972, D. P. Wojcik, *Sabal palmetto* flower stems [FSCA]

- Taylor Co., Perry, 28-IV-1970, D.H. Habeck, *Sabal palmetto* flower stems [FSCA]

- Wakula Co., St. Marks Wildlife Refuge, 28-II-1973, D.  H. Habeck, Cabbage Palm flower stalk

GEORGIA:

- Chatham Co., Ft. Pulaski Nat'l Mon, 12-I-1971, DP Wojcik, *Sabal palmetto* flower stalks [FSCA]

- Glynn, County, St. Simons Island [MCZ]

MISSISSIPI:

- Jackson / Co. 29-I-1969 / D.H. Habeck / *Sabal palmetto* [ARCC, FSCA]

SOUTH CAROLINA:

- Charleston Co. Folly Beach / 17-IX-1971, D.H. Habeck / *Sabal palmetto* flower stalk [ARCC]

- Charleston Co., Foly Beach, 17-IX-1971, D.H. Habeck, *Sabal palmetto* flower stalk [FSCA]

- Horry Co., Myrtle Beach, 27-VIII-1975, D.H. Habeck, *Sabal palmetto* flower stalk [FSCA]

Other Material Not Examined. In Dr. Dale Habeck’s files at FSCA, a number of other specimens were listed from various localities. Unfortunately, the voucher specimens for these records have not been located. However, the authors agree that because these records are within the distribution established by voucher specimens available for study, and Dr. Habeck could easily identify *Brachypeplus glaber*, these records are valid and worth publishing.

All specimens were collected in the sheaves of flower stalks of *Sabal palmetto.* Initials of collectors are DHH = Dale H. Habeck; DPW = Daniel P. Wojcik; JLG = Judy Gilmore; JLO = Jim Overman. Other abbreviations: A=adults; P=pupae; L=larvae; N=numerous, i.e. 30+ specimens were collected. Our notes are added in brackets.

FLORIDA:

- Alachua Co., Gainesville, 14-III-1968, DHH (30A)

- Alachua Co., Gainesville, 3-IV-1968, DHH (24A)

- Alachua Co., Gainesville, 10-V-1968, DPW (1A)

- Alachua Co., Paynes Prairie, 15-IX-1986, DHH, JLG, A-4056 (1A)

- Cross Creek, 23-IV-1968, DPW & DHH (10A, NL)

- Baker Co., Mcclenny, 28-VII-1969, DPW (1A)

- Bay Co., Panama City, 15-III-1971, DPW, (1A, 4 dead P)

- Brevard Co., Floridana Beach, 10-X-1969, JLO & DPW (1A, 4L)

- Charlotte Co., Murdock, 26-IX-1969, DHH & DPW (12A, 1L)

- Citrus Co., Hernando, 26-IX-1969, DPW & DHH (NA, P, L)

- Citrus Co., Ozello, 4-IX-1986, J. Gillmore & J. Watts (NA)

- DeSoto Co., Nocattee, 26-IX-1969, DPW & DHH (NA, L)

- Duval Co., Baldwin, 25-III-1969, DPW (16A, 1 callow A, NP, L)

- Flagler Co., Marineland, 19-X-1969, DHH (8A, 1P, NL)

- Franklin Co., Alligator Point, 28-XI-1975, DHH [no specimen count]

- Gadsden Co., Hinson, 18-XI-1975, DPW [no specimen count]

- Glades Co., N. of Indian Prairie Canal on SR 7, 10-X-1969, JLO & DPW (NA, P, L)

- Hardee Co., Zolfo Springs, 26-IX-1969, DHH & DPW ( ? )

- Hendry Co., Clewiston, 10-X-1969, DPW & JOL (NA, P, L);

- Hernando Co., 0.5 mi. N. of Pasco Co. line on US301, 26-IX-1969, DPW & DHH (1A, 2P, NL)

- Highlands Co., Avon Park, 26-IX-1969, DPW & DHH (NA, P, L)

- Hillsborough Co., Hillsborough River State Park, 26-IX-1969, DPW & DHH (3A, 1P, NL)

- Indian River Co., Vero Beach, 10-X-1969, DPW & JLO, (4A)

- Lake Co., Leesburg, 26-IX-1969, DHH & DPW (NA, P, L)

- Lake Co., Minneola, 26-IX-1969, DPW & DHH (13A, NL)

- Levy Co., Chiefland, 26-III-1968, DHH (1A, 1P skin, 1L)

- Levy Co., 1.5 mi. N. of Inglis, 17-IX-1986, DHH & JLG [no specimen count]

- Levy Co., 0.5 mi. E. Lebanon Station, 17-IX-1986, DHH & JLG, A-4054 (NA)

- Manatee Co., Ellenton, 26-IX-1969, DHH & DPW (9A, 1P, NL)

- Marion Co., near Grahamsville, 5-XI-1975, DHH [no specimen count]

- Marion Co., Ocala, 23-IV-1968, DHH & DPW (7A, NP, NL)

- Martin Co., Port Mayaca, 10-X-1969, JLO & DPW (13A, NL)

- Nassau Co., Callahan, 28-VII-1969, DPW (7A, 2 callow A, NP, L)

- Okeechobee Co., Ft. Drum, 10-X-1969, DPW & JLO (NA, L)

- Orange Co., 1 mi. W. of Brevard Co. line on SR 520, 10-X-1969, JLO & DPW (NA, L)

- Palm Beach Co., Canal Point, 10-X-1969, JLO & DPW (NA, P, L)

- Pasco Co., Dade City, 26-IX-1969, DPW & DHH (NA, P, L)

- Polk Co., Haines City, 26-IX-1969, DHH & DPW (8A, 1 callow A, NL)

- Putnam Co., Orange Mills, 12-VII-1969, DPW (6A, 7L)

- St. Johns Co., Hastings, 12-VII-1969, DPW (1A, 2P)

- St. Lucie Co., Pepper Park on US A1A, 10-X-1969, DPW & JLO (NA, L)

- Santa Rosa Co., Gulf Breeze, 15-III-1971, DPW (NA)

- Sarasota Co., Sarasota, 26-IX-1969, DHH & DPW (9A, NL)

- Seminole Co., Chuluota, 10-X-1969, DPW & JLO (NA, P, L)

- Sumpter Co., shore of Withlacoochee River on SR 41, 26-IX-1969, DHH & DPW (3A, 1P, 1L)

- Taylor Co., Perry, 28-IV-1970, DHH (1P, 1L)

GEORGIA:

- Chatham Co., Savannah, 27-VII-1968, [no collector] (many dead)

- Glynn Co., Blythe Island, 3-II-1973, DPW [no specimen count]

- Liberty Co., Hinesville, 15-I-1971, DPW (NA)

MISSISSIPPI:

- Hancock Co., Clermont Harbor, 17-III-1971, DPW [no specimen count]

NORTH CAROLINA:

- New Hanover Co., Wilmington [no additional data]

SOUTH CAROLINA:

- Charleston Co. Myrtle Beach, 10-VIII-1975, DHH (1A)
